# Determinants of WHO’s Problem Management Plus (PM+) uptake within health and integration sectors in Switzerland: a qualitative study

**DOI:** 10.1136/bmjph-2025-004226

**Published:** 2026-06-09

**Authors:** Dharani Keyan, Julia Spaaij, Matthis Schick, Richard A Bryant, Naser Morina

**Affiliations:** 1 School of Psychology, University of New South Wales, Sydney, New South Wales, Australia; 2 Department of Consultation-Liaison-Psychiatry and Psychosomatic Medicine, University Hospital Zurich, Zürich, Switzerland; 3 Black Dog Institute, Randwick, New South Wales, Australia

**Keywords:** Public Health, Mental Health, Qualitative Research

## Abstract

**Introduction:**

The prevalence of common mental disorders in resettled refugee populations is nearly three times higher than in the general population. The WHO recommends *Problem Management Plus* (PM+), a brief non-specialist delivered intervention for reducing common mental disorders. To identify key factors to optimise implementation, we conducted a qualitative cross-sectional study to investigate contextual determinants shaping PM+ implementation during a nationwide scale-up of PM+ in Switzerland.

**Methods:**

We conducted in-depth key informant interviews in the German-speaking and French-speaking parts of Switzerland (September 2023 and January 2025). Design and analysis of interviews was guided by the Consolidated Framework for Implementation Research (CFIR). Themes were added both inductively and deductively and categorised into challenges and opportunities for widespread PM+ service delivery.

**Results:**

We interviewed 30 stakeholders: 12 policymakers, 5 government leaders, 5 specialists and implementers and 8 non-specialist peer-providers. Whilst stakeholders were enthusiastic about the value of PM+, several limitations to its routine delivery were noted. These related to limited quality standards and control for non-specialist delivered interventions that prevented formal categorisation within the health system. Endorsement by professional associations including best-practice standards was thought to facilitate such formalised recognition. Prioritising mental health indicators within Cantonal integration programmes was perceived to facilitate funding via the national integration agenda. All stakeholders pointed towards blended co-financing pathways to ensure the longer-term sustainability of PM+ in Switzerland.

**Conclusions:**

The routine delivery of PM+ within health and integration systems presented substantial challenges and opportunities. Longer-term sustainability of PM+ may be feasible with blended financing, tailored Cantonal health and integration agendas, and established quality control standards for PM+ delivery that include formal recognition of non-specialists within the mental health system.

**WHAT IS ALREADY KNOWN ON THIS TOPIC**

- No studies to date have used standardised implementation science methodology to prospectively assess and conceptualise contextual determinants shaping the implementation of the WHO’s lay provider led psychological intervention, *Problem Management Plus* (PM+), for refugees and asylum seekers.

**WHAT THIS STUDY ADDS**

- Using the Consolidated Framework for Implementation Research, this study provides information regarding static and dynamic health and integration system factors shaping the uptake of PM+ throughout routine services in Switzerland.
- Our study provides an in-depth qualitative analysis of policymaker, organisational management, specialist and non-specialist provider perceptions of the widespread implementation of PM+.

**HOW THIS STUDY MIGHT AFFECT RESEARCH, PRACTICE OR POLICY**

- This study shows that engaging multisectoral stakeholders collaboratively is crucial to developing targeted implementation strategies that specifically modify contextual factors within health and integration sectors.

The refugee population has more than tripled around the world in the last decade.^1^

Following exposure to varied humanitarian crises arising from violence, armed conflict and natural disasters, many millions of people have sought refuge within other countries. Resettled refugees are substantially more vulnerable to mental disorders than the general population.^2^ A recent meta-analysis indicated that the prevalence of common mental disorders (CMDs) including post-traumatic stress disorder (PTSD) and depression is nearly three times higher than that observed in the general population.^3 4^ Evidence suggests that these mental health problems persist several years after resettlement.^5^ For host countries, this points to a clear need to adequately provide mental health services to meet the rising mental health needs of the population. This often entails planning for the provision of mental health services in ways that reorganises available resources within existing systems of care. Importantly, the economic impacts of such planning have future implications for the integration of refugees and asylum seekers (RAS) beyond their initial period of resettlement within host nations.

Scalable psychological (or mental health) interventions have been developed to meet the needs of adversity-affected communities across both high-income countries (HICs) and low-and-middle income countries (LMICs).^6^ The WHO’s Problem Management Plus (PM+) is one of the more widely tested scalable psychological interventions for the reduction of CMDs.^7^ Over five sessions, trained non-specialist providers deliver strategies in (a) stress management, (b) problem management, (c) behavioural activation, (d) strengthening of social supports and (e) relapse prevention. PM+has demonstrated its effectiveness in individual and group formats, including through remote video teleconferencing methods.^8^,^10^ A recent systematic review and meta-analysis of PM+implementation found it to be effective in reducing distress and promoting positive mental health outcomes in varied populations exposed to distress across both LMICs and HICs.^11^ For refugee populations, PM+was found to reduce post-migration living difficulties in Switzerland^12^ and reduced CMDs in the Netherlands.^13^

Despite the enormous potential of PM+ to be embedded within existing care systems, its implementation within host country health systems has been slow. This is not surprising because the implementation of interventions, like PM+, requires consideration for a range of contextual determinants as it relates to service provision and planning across multiple levels—policy, institutional, organisational and individual levels. These relate to barriers around the political will to scale up, professional apprehension of non-specialist delivered interventions for mental healthcare, lack of coordination between non-government organisations (NGOs) and national governments, lack of sustainable funding models, and apprehension about the use of task-sharing methods to treat CMDs.^14^,^16^ There remains an information gap in relation to the static and dynamic contextual determinants shaping delivery of scalable mental health interventions. These factors relate to real-world indicators at multiple levels of host nation governments that may facilitate the routine uptake of scalable mental health interventions. We currently do not understand under what conditions and by whom existing resources can be reorganised (and/or mobilised) to accommodate the widespread implementation of PM+ within host country systems of care.

The current study is focused on filling this gap by characterising key implementation findings gathered from Switzerland’s implementation of PM+. The Scaling-up Psychological Interventions in Refugees in Switzerland (SPIRIT) project is a national-scale-up study aiming to bridge the gap in mental healthcare for refugees by recruiting trained peer providers to deliver PM+. SPIRIT was launched following the success of its predecessor project, STRENGTHS, where PM+ was found to be both clinically and cost effective in reducing distress and improving the well-being of Syrian refugees in varying countries in Europe and the Middle East.^17^ Current knowledge of barriers and facilitators of PM+ implementation remains dominated by post hoc application of frameworks and theories where there is a dearth of evidence derived from implementation science studies. To fill this gap, the current study investigated the static and dynamic determinants shaping widespread PM+ delivery in the German and French-speaking parts of Switzerland. We present the perceptions and experiences of key stakeholders from a health and integration systems perspective.

## Methods

### Study design

The cross-sectional qualitative study adheres to the consolidated criteria for reporting qualitative research^18^ and was conducted over 17 months during the mid implementation phase of SPIRIT (September 2023 and January 2025), where PM+implementation was underway across six asylum regions. SPIRIT is funded by the State Secretariat for Migration (SEM) and further information is provided in the supplement. The Consolidated Framework for Implementation Research (CFIR) (version 1 and 2)^19 20^ was used to characterise static and dynamic determinants of PM+ uptake across multiple levels of the health and integration system. To the authors’ knowledge, no studies have used the CFIR to prospectively investigate the potential for a nation-wide scale-up of PM+ in LMIC or HIC settings. Three research questions were investigated from the perspectives of health and integration sector stakeholders: (1) challenges and opportunities for PM+ introduction within the health and integration systems; (2) perceived impacts of PM+ on refugee integration and (3) the potential for nation-wide scale-up of PM+ in Switzerland.

### Study population and sampling

Study participants were identified across policy, institutional, organisational and individual levels within health and integration sectors in Switzerland. Participant selection followed an ecological framework, assuming that future integration of PM+ into existing care systems will be shaped by multilevel factors. At the individual level, this included trained peer (ie, refugee) providers delivering PM+ for RAS. At the organisational management level, it included professionals responsible for the training and coordination of non-specialist providers. At the institutional level, it included any professional, chief psychiatrist or director within the Cantonal health and integration sector engaged with evidence-based mental health programmes, past or present. At the policy level, this included government officials or policymakers whose routine work comprised decision-making on programmes for RAS within health and integration sectors.

Participant eligibility was determined fluidly through purposive and snowball sampling, with all stakeholders approached for participation via email, telephone or in-person. All invited stakeholders participated with no refusals. Data were collected through 27 individual key informant interviews and 5 focus group discussions (2–3 participants per group). Table 1 outlines distribution of participants by stakeholder group, interview format and number of data collection sessions. We recruited 12 policymakers, 5 government leaders, 5 specialists and implementers and 8 non-specialist peer-providers. The term ‘in-depth interviews’ is used to refer collectively to both key informant interviews and focus group discussions. To ensure confidentiality of data obtained, individual characteristics relating to age and gender have not been provided.

**Table 1:** Study participants interviewed by stakeholder group

Stakeholder group	Description	Total participants (n)	Key informant interviews (n)	Focus group discussions (n)	Total data collection sessions (n)
Federal health and integration policy leaders (ie, policymakers)	National health leaders within the Federal Office of Public Health, the Federal Health Quality Commission, State Secretariat of Migration and Health Promotion Switzerland	12	5	4	9
Cantonal health and integration leaders	Chief Doctors, Lead psychiatrists of refugee and asylum seeker mental health services within central and western Switzerland asylum regions, Cantonal Commissioner for Health Promotion	5	4	1	5
SPIRIT Management team	PM+ programme leader within a national NGO, Psychiatrists, Clinical psychologists, Coordinator of peer providers	5	6*	0	6
Individual providers	Trained refugee peer providers of PM+	8	12†	0	12

* The PM+ coordinator was interviewed on two occasions.

† Three peer providers were interviewed on two occasions.

NGO, non-government organisation; PM+, Problem Management Plus; SPIRIT, Scaling-up Psychological Interventions in Refugees in Switzerland.

### Procedures

The CFIR framework was systematically applied to guide design of interview guides, data collection and evaluation. First, the innovation was defined as non-specialist delivered brief mental health interventions for RAS, with PM+ as the exemplar intervention. Second, the inner setting was defined as the Cantonal institutions (ie, State institutions) coordinating PM+ implementation, with data collected from Zurich and Vaud—each represented a study site of the SPIRIT project. Third, the outer setting referred to the national Swiss context and implementation climate within which the inner setting operated. Accordingly, descriptions of the five CFIR domains were reviewed (intervention characteristics, outer setting, inner setting, characteristics of individuals and process), and relevant constructs were mapped to SPIRIT as it related to scalable psychological intervention delivery. The systems-level frame (ie, coordination through health, migration, integration sectors) led us to prioritise constructs that operated at institutional and policy levels. For this reason, CFIR domains including characteristics of individuals and implementation process were examined where they emerged organically but were not the primary analytic lens.

Initial topic guides for each stakeholder group were developed by DK and piloted via ongoing discussion with all study authors prior to formal data collection. To this end, the data collection strategy was iterative in that ongoing discussions between the first author (DK) and last author (NM) led to identification of new key informants. Additionally, interview topic guides were iteratively revised whereby debrief discussions after each stakeholder group (ie, individual and organisational management groups) led to adaptations of relevant topic guides for subsequent groups (ie, institutional and policy groups). Each interview collected information on participants’ professional background and experience, followed by open-ended questions. Interviews were led by DK and JS (both are female postdoctoral researchers with a background in clinical psychology and 5 years’ experience in conducting interviews).

In-depth interviews were conducted in English (n=22) and German (n=10). Individual key informant interviews had a target duration of 60 min (range 45–90 min) and focus group discussions a target duration of 90 min (range 60–120 min). Each in-depth interview commenced with a brief introduction by DK, an explanation of the study goals, and a request for oral consent. Field notes and regular debrief discussions between study authors (DK, JS, NM and MS) ensured collaborative data interpretation driven by local needs. Thematic saturation was assessed across the full sample and considered reached when no new CFIR constructs were emerging within specific domains across successive interviews. Stakeholder feedback was triangulated with seven supplementary source documents to verify accuracy and support thematic saturation in that no new domain-relevant themes were identified. These source documents comprised of policy briefs and government documents on RAS service provision and planning and were nominated by stakeholders. To validate extracted themes, preliminary findings were presented to SPIRIT stakeholders at dissemination meetings for member checking.

### Data management and analytic workflow

All interviews were audio-recorded with informed consent, pseudonymised, and transcribed via *Rev.ai*, an online Artificial Intelligence-based tool (95% accuracy in English). German transcripts were checked for accuracy by JS and NM. Verified transcriptions were initially coded by DK using thematic content analysis guided by the CFIR framework in NVivo V.14, with passages as the unit of coding. JS independently verified a subsection of DK’s coding with disagreements resolved through discussion, and NM and RAB consulted where consensus could not be reached. Coding proceeded both deductively, guided by three primary CFIR domains selected a priori, and inductively, where constructs from the Characteristics of Individuals and Process domains emerged organically and were incorporated accordingly. Each construct was classified as a barrier, facilitator, or both in relation to PM+ service delivery. A full coding tree is provided in the online supplemental materials.

### Reflexivity and positionality

The research team occupied varying insider and outsider positions relative to SPIRIT warranting transparent reflection. DK led all interviews as an outsider to the Swiss implementation context, with no direct involvement in SPIRIT, though a background in implementation science may have introduced assumptions about meaningful implementation barriers in high-income systems. JS and NM held substantive insider positions; NM as principal investigator of SPIRIT and JS as supervisor of the peer providers interviewed, creating a power asymmetry that may have constrained peer provider willingness to voice criticism. To mitigate this, all peer provider interviews were conducted by DK and a Masters student proficient in Swiss German, with JS and NM intentionally excluded. The team’s broader institutional proximity to SPIRIT may have shaped interpretation of ambiguous findings. This was mitigated through independent coding, disagreement resolution with NM and RAB, and member checking at SPIRIT dissemination meetings. We present these considerations not as invalidating the findings, but as necessary context for their interpretation.

## Results

**Figure 1:**
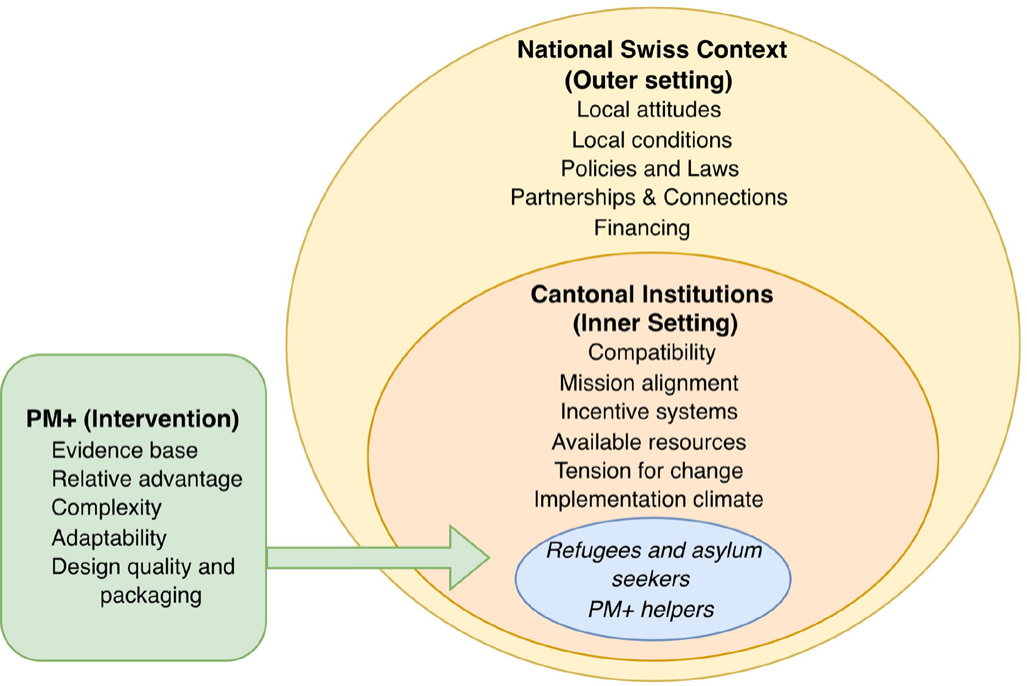
Examining determinants of PM+ implementation in Switzerland using the Consolidated Framework for Implementation Research. PM+, Problem Management Plus.

30 participants completed in-depth interviews (12 national policy leaders, 5 institutional leaders, 5 organisational management team members and 8 peer providers; see table 1). Findings are organised by CFIR domain in table 2 (see figure 1). Illustrative quotes are presented in table 3, selected on the basis of clarity of expression and breadth of stakeholder representation,^21 22^ reflecting perspectives consistently expressed across multiple participants and data collection formats.

**Table 2:** Summary of key challenges and opportunities to wide-spread PM+ implementation, by CFIR domain, as reported in in-depth stakeholder interviews and documentation review

CFIR domains	Constructs	Challenges (data source)	Opportunities (data source)
Intervention characteristics (ie, PM+ as the exemplar scalable psychological intervention)	Relative advantage Evidence base Complexity Adaptability Design quality and packaging	Need for localised evidence on the effectiveness of PM+ (Federal health policy leaders, Cantonal psychiatrists) PM+ is a novel service category not exclusively aligned with health, integration or social initiatives (Federal and Cantonal leaders) Lack of formal titles or recognition of the helper creates confusion and undermines longer-term legitimacy of the role (Federal Quality Commission, peer providers)	Evidence of relative advantage in relation to available services for RAS (all stakeholder groups) Establishing accreditation frameworks and quality control guidelines (eg, minimum qualifications, supervision protocols, outcome tracking) could make PM+viable within formal systems of care (eg, health) (Cantonal Health leaders, Federal leaders)
Outer setting (ie, National Swiss context)	Local attitudes Local conditions Policies and Laws Financing Partnerships and connections	Lack of national health policy on the role of non-specialist/peer-delivered initiatives for mental healthcare (Federal Health leaders, Doc) Decentralised governance of health leads to variation in initiatives for RAS across Cantons (IDI, Doc) Mental health screening is not mandated by law at the Federal asylum centres (Federal and Cantonal integration leaders, Doc) Professional scepticism with public health (eg, psychiatry) on the role of PM+ in adequately supporting RAS (reported secondhand by Cantonal Health Leaders, Federal Leaders) No established financing pathway for non-specialist delivered interventions (Doc, all stakeholder groups)	Cantons can lead ‘bottom-up’ efforts to prioritise mental health and advocate for Federal alignment (eg, budget support via SEM) (Cantonal Health and Integration leaders) If screening measures were put in place at the Federal centres, and this was shared with Cantons, it could aid service-planning for longer-term integration support (Cantonal health leaders) Public health officials need education on the ways in which PM+ can be incorporated as a low-threshold initiative (eg, collaborative care, stepped care pathways) (Cantonal and Federal leaders) The FOPH mandate to promote health equity offers justification for system-level support of PM+ (Federal Health leaders, Federal Quality Commission, Doc) Establishing best-practice guidelines through professional associations that included non-specialist delivered interventions can help inform enforceable standards at the Federal level (Federal leaders) Blended financing through State Secretariat of Migration, Health Promotion Switzerland and Federal Health needs exploration (all stakeholder groups, Doc)
Inner Setting (ie, Cantonal Institutions)	Compatibility Mission alignment Incentive Systems Available resources Tension for change Implementation climate	There is a lack of formal guidelines on where and how PM+fits in within Cantonal health and integration systems (Cantonal Psychiatrists) Equity in health service provision to RAS is not formally measured (Doc, Federal Quality Commission, Federal Health leaders) Prioritisation of PM+ as a service for refugees varies across Cantons according to individual priorities (Cantonal Health and Integration leaders)	PM+ was perceived to align with other integration initiatives (eg, job coaching, language lessons) in promoting Cantonal integration goals (Cantonal Integration leaders) Professional associations (eg, psychiatry, clinical psychology) can advocate for PM+ with policy decision-makers (Federal and Cantonal Health leaders) Development of specific health equity indicators for mental healthcare (eg, access to interpreters) can create a pathway for PM+ accessibility (Federal leaders)

CFIR, Consolidated Framework for Implementation Research; Doc, documentation review; FOPH, Federal Office of Public Health; IDI, in-depth interview; PM+, Problem management Plus; RAS, refugees and asylum seekers; SEM, State Secretariat for Migration.

**Table 3:** Interview quotes organised by CFIR domains

Domains	Quotes
Outer setting	“*I think the legal issue [referring to lack of national health policy mandate] is not helping us. The finances are not helping us… For us to become active, what needs to happen, we need a demand from a parliamentary group, which we will answer to… then we will have the political mandate to act” –* National health leader
	*“There’s no, um, policies regulating health equity. It’s being, uh, put up on the agenda just quite recently… Our side has been, uh, being talked about as health inequalities for a number of years and just being mentioned, you need to have it there. And now we're talking about health equity…And also when it comes to prevention of mental health, we had, we don't have a specific plan yet”* – National Health leader *“First of all, but this, I think it’s always, uh, obstacle, this always, uh, professional, uh, uh, opposition. And this, I think from psychiatric, because Switzerland, like many accidental countries, uh, psych mental health is hospital centric, psychiatric centric, let’s say, call it like that*” - Cantonal Health leader
Intervention	“*Sanatorium XX, for example, is a large psychiatric care facility in our Canton. They are very progressive in this regard and started involving peers relatively early on. And they always report very good results.”* – Cantonal Health Leader “*From my perspective, it’s a newly defined role. I mean, a psychologist, a psychiatrist has a clear role. A counsellor has a clear role. A nurse has a clear role. But a helper, when I say helper, they first ask me, oh, are you not getting paid? Is it voluntary? And then I say, no, I am getting paid.”* – Peer provider
	*When you have science and you look at the system…you need like, accreditation of this peer based [model of care*]. *That you have a label”* -National Health leader
Inner setting	“*It’s not to put the mental health before and then integration. It’s how we can manage both together*” – Cantonal integration leader
	*“Firstly, we obviously have a serious shortage of skilled workers and we don't have enough specialists to cover everything. In that respect, I think peers are a very good thing”. –* Cantonal Health leader *“At the beginning it* (referring to Cantonal Integration Programs—KIP) *was a program only for social institutions. But in Canton Vaud, we introduced the health. And especially the mental health, because we know it’s very clear. If you want to integrate someone to, to learn the language To, to find a job. Uh, mental health is crucial”. –* Cantonal Health leader

CFIR, Consolidated Framework for Implementation Research.

### Characteristics of the PM+ intervention (CFIR domain 1)

The following factors were characteristics of PM+ that influence its appeal as a (mental) health and/or integration initiative and in turn the likelihood of its successful future integration.

#### Relative advantage

All stakeholders were enthusiastic about the potential of PM+ as a first-line mental healthcare service for RAS. NGO leaders, non-specialist providers and Federal health policy leaders noted its marked advantage over specialist services given the absence of comparable initiatives for refugees in Switzerland. Key advantages included peer providers sharing the same language as beneficiaries, fostering trust and authenticity, and serving as a positive first contact with the health system that could increase future engagement with specialist care.

#### Evidence strength and quality

Federal integration and health policy stakeholders stated localised evidence for the role of PM+ in preventing CMDs and influencing social and integration outcomes in Switzerland was insufficient. They expressed a desire to see the extent to which outcomes of SPIRIT supported these purported claims for PM+. Cantonal health leaders expressed an openness for peer-based delivery of PM+ and cited similar examples of this model of care that currently operated within other psychiatric services (ie, substance misuse and addiction care). Health promotion stakeholders (see supplement for definition) similarly recognised use of peers as a first-line level of support and referred to parallel initiatives that currently existed for refugees (ie, ‘Bridge builders’ programme).^23^

#### Complexity and adaptability

Cantonal stakeholders noted that PM+ was perceived as a novel service category in that all stakeholders struggled to categorise it as an integration, health, social welfare initiative or some combination of all. This lack of categorisation made it difficult to integrate PM+ within existing systems of care (ie, health and/integration systems). For example, Cantonal health leaders expressed concern over siloing PM+ as just an integration initiative as this would likely limit where it could be delivered (eg, such as primary healthcare settings). At the same time, it was unclear whether PM+ was preventative in that it could, for example, reduce avoidable hospitalisation rates. The confusion over its role insofar as reducing CMDs, promoting social and/or economic integration was thought to hinder the buy-in from other specialist stakeholders including psychiatrists and physicians. For example, Cantonal psychiatry representatives noted that their respective colleagues were unclear whether PM+ was designed to replace specialist interventions for RAS. It was also unclear at what stage of the asylum process that PM+ should be prescribed to RAS.

#### Design quality and packaging

Perceptions on how PM+bwas packaged in terms of its usability and design converged across stakeholders. First, ‘helpers’ (ie, refugee peer providers of PM+), as they are currently referred to within the SPIRIT project, expressed difficulties with explaining the nature of their role to beneficiaries and (mental) health specialists outside of the SPIRIT project (see table 3—intervention quotes). The lack of a formalised title for this role was perceived to somewhat undermine the acceptability of their role as a non-specialist provider of mental healthcare. Cantonal health leaders expressed the need for some form of certification (or accreditation) standards to be assigned to the role of a PM+ helper. To this end, refugee peer providers expressed that the lack of accreditation pathways limited their professional identity. Policymakers expressed that the absence of standardised inclusion/exclusion criteria for recruitment of helpers would make quality assurance and financing more challenging in the future. To this end, they expressed a need for clear monitoring and evaluation standards for ongoing PM+ delivery. Health leaders and policymakers expressed that PM+could be viable within existing systems of care (eg, health system) if quality assurance and accreditation challenges are addressed (eg, minimum qualifications, supervision protocols, outcome tracking). Federal health and integration leaders expressed the potential for PM+ to be repositioned beyond the migrant and asylum context to other vulnerable groups (eg, prisoners, health workers, schools), thus widening its relevance to policymakers.

### National Swiss context (CFIR domain 2: outer setting)

The ‘outer setting’ domain summarises external factors shaping PM+ implementation, including local attitudes and conditions toward refugee needs at a national level, relevant policies and laws, and how funding guidelines are expected to impact PM+ service delivery.

#### Local attitudes and conditions

Swiss integration programmes under the State SEM prioritise practical integration (eg, language, employment) with little attention to mental health. Decentralisation of health, where Cantons autonomously implement varied initiatives leads to inconsistencies across different asylum regions. At the same time, Cantonal health leaders argued it as an opportunity for PM+ to be adopted in a tailored way, especially as RAS transition into Cantonal care. To this end, policymakers and Federal public health stakeholders advocated for sharing mental health screening outcomes with Cantons (following asylum procedures at Federal centres) to better align with Cantonal service planning for long-term integration support. Health leaders and policymakers expressed that professional scepticism for PM+ remains, with some perceived to doubt that peer-delivered care sufficiently meets the mental health needs of RAS. Consequently, they expressed the importance of educating specialists (eg, psychiatrists) and public health stakeholders on what ‘low-intensity’ (or low-threshold) meant in practice, and how non-specialist peer-delivered care can operate within structured models (eg, stepped care or collaborative care) without compromising the quality of care. To this end, Cantonal health representatives acknowledged workforce shortages and perceived PM+ as a potential ‘gap-filler’.

#### Policies and laws

Mental health screening is not mandated at Federal asylum centres. Federal integration leaders expressed concerns with introducing mental health screenings (eg, for depression, PTSD) as was perceived to complicate and potentially interfere with asylum procedures at Federal centres. This discourages a centralised screening mandate and limits Federal level implementation of PM+. Cantonal health leaders argued that the lack of a national health policy that regulated how non-specialist (or peer-based) mental healthcare should be delivered created uncertainty for PM+integration within existing funding structures. Nevertheless, Federal Quality Commission (see supplement for definitions) stakeholders argued that the Federal Office of Public Health’s (FOPH) legal mandate to promote health equity offered a potential entry point for justifying system-level support for PM+ (see table 3—outer setting quotes). To explore this path, they called for measurable (mental) health equity indicators that currently did not exist.

#### Partnerships and connections

Switzerland’s decentralised governance limits coordination across Federal and Cantonal institutions, and integration and health sectors. NGO, health and integration leaders argued that this limited cross-sector coordination of mental health services for RAS. For example, Cantonal health leaders stated that a routine referral pathway from Federal asylum centres to Cantonal mental healthcare systems did not exist. This was related to a lack of mental health screening at Federal centres. To this end, Federal and Cantonal stakeholders stated that Cantons could advocate for a centralised screening strategy or similar mandates as this served as a structural bridge between Federal and Cantonal systems.

#### Financing

Stakeholders agreed that no established funding model exists for scalable, non-specialist delivered interventions. Current mandatory health insurance funds limited forms of specialist care (ie, psychotherapy delivered by psychologists) for CMDs. The viability for blended financing by combining resources from health, integration, and prevention sectors was explored. The potential for the FOPH to support financing of PM+through its health equity mandate was floated by Health Promotion stakeholders. Federal health and integration leaders argued that establishing best practice guidelines (eg, through Swiss professional psychiatry associations) that included non-specialist delivered care can lead to enforceable standards under the health sector (eg, FOPH), but this required partnerships between professional associations and Federal public health entities. Health leaders argued that co-financing PM+ under Cantonal Integration Programmes (KIP)^24^ was an option given that Cantons defined integration priorities under the federal mandate of integration. To this end, the inclusion of mental health as part of their KIP outcomes, Canton Vaud had created a pathway for incentivisation of PM+ type services under the national integration agenda. Finally, funding through Health Promotion pathways was argued as a potential path if PM+ was found to be preventative in reducing the risk of CMDs. To this end, mandatory health insurance could be leveraged as partial funding for PM+ as funding for complementary medical services (eg, physiotherapy) currently exists under this pathway.

### Cantonal settings (CFIR domain 3: inner setting)

The ‘inner setting’ examines the implementation climate and preparedness for PM+ service delivery within routine service provision pathways.

#### Compatibility

Health and integration leaders perceived PM+ to fit within a tiered or stepped care model with the health system (eg, prior to specialist support). Integration leaders perceived an alignment of PM+ with other integration services, where it had the potential to increase engagement with related supports (eg, job coaching, language lessons). Canton Vaud Health representatives expressed a strong confidence in PM+ as a viable service for refugee populations, in the absence of clear alternatives. Cantonal integration leaders emphasised the need for positioning PM+ within a preventative frame as this was thought to increase its strategic fit within the integration agenda, as well as potentially aligning indicators with the FOPH mandate for promoting health equity.

#### Mission alignment

All Cantonal leaders agreed that PM+was not easily categorised as solely a health or integration initiative, and this created a challenge for institutional alignment and funding within Cantonal systems. While PM+ conceptually aligned with the Federal health equity mandate, stakeholders were unclear on specific guidelines relating to where and how PM+ fits in. Federal health leaders emphasised a need for clinical leadership (eg, professional psychiatry associations) to advocate for PM+ with policy decision-makers. To do this, it was recognised that educating clinical leadership on the proposed role of PM+ as complementary, rather than competing with specialist care, could ease their resistance and increase motivation for advocacy efforts. This was emphasised as an instrumental step in mobilising longer-term resource planning beyond project-based funding. For PM+ to be meaningfully integrated within Cantonal systems of care, health and integration leaders emphasised the need for routine mental health screening, followed by immediate access to low-intensity interventions like PM+.

#### Incentive systems and available resources

Equity in health service provision to RAS is not measured (see table 3). Here, developing indicators of health equity (eg, access to interpreters) will create an avenue to prioritise accessibility of PM+ to RAS. Health leaders argued that the lack of systematic funding for professional interpreters (eg, Canton of Zurich) points to unequal access of (mental) health services. To this end, investing in professional interpreter services was viewed as a foundational step towards equitable (mental) healthcare and broader sustainability of PM+. Health leaders emphasised that indicators of health equity should be developed and prioritised by Cantons. For example, this could include linking access to interpreters for RAS with measurable health equity goals (eg, reduced inequalities in access to health services).

#### Tension for change and implementation climate

Health leaders noted that the priority for PM+implementation varies across Cantons, and this is related to refugee population size across Cantons. To this end, mental health had yet to be embedded in many Cantonal integration agendas, and this currently limited opportunities for PM+ implementation. Canton Vaud was the first to institutionalise mental health as part of its KIPs, and this offered a model for other asylum regions around the country. Canton of Zurich receives approx. 18% of RAS,^25^ and this is leading to growing internal pressures to coordinate and invest in mental health initiatives. Health and integration leaders acknowledged an increasing awareness of the need to support the mental health and integration of RAS in parallel, and this likely creates an opening to align PM+ within their respective sectors (see table 3—inner setting). Here, NGO and integration leaders strongly viewed PM+ as a critical intervention for RAS given a lack of alternatives. At the same time, formal recognition of non-specialist delivered interventions by Federal and/or Cantonal health directorates was argued to be essential for long-term sustainability. For health directorate endorsement (ie, FOPH), policymakers emphasised the need for clear operational standards for PM+ implementation (ie, quality assurance standards) and that this was a major barrier to securing sustainable funding in the longer-term.

### Secondary analyses

A cross-cutting pattern emerged across the intervention characteristics, outer setting and inner setting domains whereby perceived professional resistance may be characterised as a recursive implementation barrier. As one Cantonal health leader observed, professional opposition to task-sharing models was a predictable feature of hospital-centric and psychiatry-centric systems, where the boundaries of mental healthcare have historically been governed by specialist institutions (see table 3—quote 3). Notably, this resistance was reported secondhand by participants, rather than expressed directly, mapping inductively onto the individual characteristics domain construct of individual stage of change. This perceived resistance connected directly to the complexity and design quality constructs, whereby the absence of accreditation frameworks and formalised recognition for PM+ helpers meant professional organisations lacked a shared basis for evaluating PM+ within existing care hierarchies. Within the inner setting, this surfaced in the mission alignment and incentive systems constructs, where professional association endorsement was identified as a prerequisite for formalised Cantonal funding, yet remained contingent on underdeveloped quality and accreditation standards. This may be characterised as a recursive pattern where absent professional endorsement constrained quality standards development, which in turn limited formal recognition and sustainable funding.

Inductively, findings also mapped onto the CFIR Process domain constructs of planning and engaging. Federal Quality Commission, FOPH and Health Promotion stakeholders converged on the health equity mandate as the most viable institutional entry point for championing PM+, reflecting prospective planning thinking about how existing policy levers could be mobilised to drive implementation forward institutionally.

## Discussion

The current study provides key insights into the challenges and opportunities for systematically implementing PM+ through health and integration systems in Switzerland. To our knowledge, this is the first study to prospectively apply the CFIR^19 20^ to understand system-level barriers and enablers to PM+implementation, especially within a decentralised host nation setting. While many challenges identified have been previously reported (eg, professional scepticism, lack of sustainable funding pathways),^14 15^ the current study reveals important unanticipated considerations for PM+ implementation within Swiss health and integration sectors.

Framed through the intervention characteristics domain, all leaders, providers and policy decision-makers readily acknowledged the relative advantage of PM+ as an important first-line intervention to support refugee mental health and their integration within Cantons. At the same time, the need for an evidence base that was localised to Switzerland insofar as the potential early interventionist role of PM+ in facilitating refugee integration was highlighted by policy decision-makers. Despite the potential for PM+ as a low-threshold mental health initiative, several Cantonal health leaders described professional scepticism that prevented formal endorsement. This related to the perceived unclear objective of PM+ in terms of whether it competed with specialist care services. To this end, it was acknowledged that formal mental health associations and leading psychiatry representatives required education on how PM+ could be embedded within stepped care and/or collaborative care initiatives for RAS. The nature of this professional scepticism warrants careful interpretation. As scepticism was reported secondhand by study participants, the current study cannot disentangle whether resistance reflects primarily a knowledge deficit, whereby specialists lack sufficient familiarity with task-sharing models, or a structural concern rooted in jurisdictional boundary protection and implications for reimbursement structures within Switzerland’s mandatory health insurance system. Both interpretations are plausible and are not mutually exclusive. While federal and cantonal health leaders framed this scepticism as a knowledge deficit and in turn highlighted the need for best-practice recommendations, we recognise that this potentially underestimates the structural dimensions of professional opposition within psychiatry. Future research that directly engages sceptical specialist stakeholders would be necessary to disentangle these competing explanations. Integration and health leaders put forth a need for strategic positioning of PM+ as a low-threshold preventative service that complemented, rather than replaced specialist mental healthcare. It is worth noting that this preventative framing was identified as a strategic deployment of language to facilitate institutional embedding and financing access, rather than a clinical reclassification of PM+. It remains that PM+ is an indicated early intervention targeting individuals experiencing clinically significant distress. Future implementation efforts should be careful to maintain this distinction in stakeholder communications to avoid conceptual ambiguity about PM+ and its appropriate placement within stepped models of care.

The most concrete barrier to systematic PM+ implementation in Switzerland was structural characteristics of the Swiss setting involving its siloed governance of health, welfare and integration systems as it pertained to refugee and asylum seeker care within Cantons. Specifically, the lack of a unified mental health strategy for RAS arriving in the country impacts the extent to which Federal health entities (eg, FOPH) can legally enforce coordination of mental health services for RAS within Cantonal institutions. Here, the urgency to prioritise mental health support was thought to vary across the 26 Cantons, and this was likely shaped by refugee intake and perceived tension for change in meeting the unmet needs of the population. One Canton provided an exemplar of high implementation readiness where it had advocated for mental health support as part of its individualised KIP. In turn, this had opened a co-financing pathway through existing integration programmes. While co-financing of PM+ under the KIP represents a pragmatic financing pathway, routing refugee mental healthcare through integration rather than health equity budgets risks framing refugee mental healthcare primarily as an integration challenge. A critical examination of what this structural routing means for the long-term governance and equity of refugee mental healthcare in Switzerland warrants attention in future policy and implementation research. Further, PM+ was perceived as a novel service category (ie, design quality and packaging) and this in part was due to the lack of formalised recognition of the role of PM+ ‘helpers’ and limited formal guidance on use of non-specialist interventions for refugee mental healthcare. This importantly made service planning within existing care pathways of health and/or integration a challenging pursuit. Consistent with past evidence,^15 16^ stakeholders reiterated that PM+ did not readily fit within mandatory health insurance service provisions. To this end, the absence of quality control standards for PM+ service delivery was a considerable barrier for its resource allocation within mandatory health insurance.

Given the absence of an established financing pathway for non-specialist delivered interventions for refugees, co-financing emerged as a prominent theme across the financing and partnerships and connections constructs. PM+’s high compatibility with existing integration initiatives (including job coaching, language lessons and social support) was seen by integration leaders as justifying its co-financing within the broader integration agenda. Policymakers and health leaders identified the FOPH health equity mandate as a pragmatic co-financing entry point, whereby Federal incentivisation of Cantons to meet health equity goals could create a pathway for longer-term PM+ sustainability within the health system. However, stakeholders agreed that national best-practice guidelines and accompanying quality standards, including supervision, evaluation and accreditation frameworks, were prerequisites for formal funding through mandatory health insurance. Finally, mandating mental health screening at Federal centres or Canton entry was advocated as a structural prerequisite for PM+ referral pathways, consistent with recommendations from the National Commission for the Prevention of Torture for early identification of vulnerable persons within reception centres.^26^

### Implications for policy and practice

While a systematic mapping of implementation strategies was beyond the scope of the current study, findings tentatively point toward several evidence-based implementation directions, drawing broadly on existing implementation strategy compilations such as the ERIC framework.^27^ First, a coordinated coalition across key Federal and Cantonal funding bodies, namely the State Secretariat for Migration, the FOPH and Health Promotion Switzerland could provide a concrete structural pathway for co-financing mental health initiatives alongside existing integration priorities. Second, the absence of mental health equity indicators, and for refugee mental healthcare, points to a need for developing measurable equity metrics. These could include interpreter access rates and uptake tracking of referral pathways that incorporate non-specialist delivered care. Third, addressing professional scepticism toward task-sharing models may benefit from a dual strategy of preparing institutional champions within psychiatric and public health leadership to advocate for non-specialist delivered care, alongside the adoption of an established quality monitoring framework such as the WHO’s EQUIP toolkit,^28^ which provides internationally recognised standards for supervising and evaluating non-specialist mental health providers. Together, these directions are offered as tentative practice recommendations to inform future implementation planning rather than as prescriptive strategies, and each would require co-design with relevant stakeholders, piloting and evaluation before adoption within the Swiss context. Finally, the determinants identified here, particularly the interdependence of accreditation standards, professional endorsement and blended financing, may offer a practical diagnostic framework for initiating comparable PM+ implementation efforts in other high-income federal systems. Countries such as Germany, France and Belgium share Switzerland’s decentralised governance and mandatory health insurance structures, though their integration policy landscapes differ, making them natural next contexts in which to test whether these determinants generalise or whether country-specific adaptations are required.

### Limitations

Our study had several limitations. We intentionally sampled stakeholders from German-speaking and French-speaking regions where active motivation to sustain PM+ beyond SPIRIT had been expressed, introducing a systematic bias toward high implementation readiness contexts. Findings should therefore be interpreted as reflecting the implementation landscape within motivated Cantons rather than the broader Swiss context. Cantons with lower refugee intake, limited PM+exposure or weaker cross-sector coordination may present a qualitatively different implementation landscape, and future research should deliberately sample from less implementation-ready Cantons to characterise the full range of determinants shaping PM+ uptake. While interviews were conducted within official settings independent of SPIRIT activities, the uniformly positive stakeholder tone may partially reflect social desirability effects, notwithstanding written assurances of anonymity provided to all participants. Additionally, all non-English speaking participants were proficient in English and offered the opportunity to respond in German or French respectively. At the same time, we acknowledge that conducting interviews in a second language may have limited the depth or nuance of expression for some participants. Further, interview guides were not designed to address all CFIR constructs, where missing constructs reflect study protocols rather than an indication of their irrelevance to future systems-level implementation research. Finally, while non-specialist peer providers were themselves from a refugee background, the perspectives of PM+ recipients as clients were not captured in this study, and future research should deliberately incorporate beneficiary voices to ensure implementation strategies are responsive to the needs and experiences of the target population.

## Conclusions

Notwithstanding these limitations, findings may offer tentative insights for other high-income systems characterised by decentralised governance, siloed health and welfare systems, and the absence of a unified national mental health strategy for refugee populations; structural features that are shared by countries such as Germany, Canada and Australia. A detailed comparative analysis across federal contexts is beyond the scope of the current study but represents a valuable direction for future implementation research. To our knowledge, this is the first study to prospectively apply the CFIR to examine system-level determinants of PM+implementation in a high-income host nation, extending previous qualitative evidence on barriers to non-specialist delivered interventions by incorporating policy, institutional and health leader perspectives. Together, findings provide a necessary foundation for developing targeted implementation strategies for widespread PM+ delivery in Switzerland.

## Supplementary material

10.1136/bmjph-2025-004226 online supplemental file 1

## Data Availability

Data sharing not applicable as no datasets generated and/or analysed for this study.
