# Supplementary material for: Determinants of WHO’s Problem Management Plus (PM+) uptake within health and integration sectors in Switzerland: a qualitative study
Source: BMJ Public Health. 2026 Jun 9;4(2):e004226. doi: 10.1136/bmjph-2025-004226 (PMC13264938; doi:10.1136/bmjph-2025-004226)
Supplement: online supplemental file 1 [file bmjph-4-2-s001.docx]

Table of Contents

[Table of Contents 1](#_Toc223368568)

[SPIRIT Project 2](#_Toc223368569)

[COREQ (COnsolidated criteria for REporting Qualitative research) Checklist 3](#_Toc223368570)

[Interview topic guides 5](#_Toc223368571)

[Policy makers 5](#_Toc223368572)

[Specialists and SPIRIT organisational management 7](#_Toc223368573)

[Non-specialist providers 10](#_Toc223368574)

[Coding: CFIR constructs and definitions 13](#_Toc223368575)

[Study groups overview 15](#_Toc223368576)

[Coding tree 16](#_Toc223368577)

# **SPIRIT Project**

SPIRIT is funded by the Prevention in Healthcare project of Gesundheitsförderung Schweiz (Health Promotion Switzerland), whereas the single Cantonal implementing partners are financed through the State Secretariat for Migration (SEM) in all six asylum regions, and respective Cantonal authorities. The Prevention in Healthcare Project supports innovative interventions in key areas of non-communicable diseases (NCDs), addiction and mental illness. The aim is to increase preventative approaches to healthcare within the wider Swiss health care system. The SPIRIT project aims to reach 2000 refugees across varied cultural and linguistic backgrounds.

The French-speaking (Canton Vaud) part is supported by Bureau cantonal de l’Intégration (Canton Office of Integration), Département de psychiatrie du CHUV (Department of Psychiatry of the Canton Vaud), and Direction Générale de la Santé (Directoralte of General Health). The PM+ intervention is offered to refugees of varied backgrounds including Arabic, English, Farsi/Dari, French, Kurdish, Russian, Tigrinya, Turkish, Ukrainian within Canton Vaud.

In the Canton of Zurich, SPIRIT is additionally financially supported by Fachstelle Integration (Canton of Zurich Integration department), and Gesundheitsdepartement Zürich (Zurich Department of Health). The PM+ intervention is offered to refugees of varied backgrounds including Arabic, German, English, Farsi/Dari, French, Kurdish, Pashto, Russian, Tamil, Tigrinya, Turkish, Ukrainian within Canton Zurich.

# **COREQ (COnsolidated criteria for REporting Qualitative research) Checklist**

| **Topic** | **Item No.** | **Guide Questions/Description** | **Reported on Page No.** |
| --- | --- | --- | --- |
| **Domain 1: Research team and reflexivity** | | | |
| *Personal characteristics* | | | |
| Interviewer/facilitator | 1 | Which author/s conducted the interview or focus group? | 8 |
| Credentials |  | What were the researcher’s credentials? E.g. PhD, MD | 1, 8 |
| Credentials |  | What was their occupation at the time of the study? | 8 |
| Gender |  | Was the researcher male or female? | 8 |
| Experience and training |  | What experience or training did the researcher have? | 8 |
| *Relationship with participants* | | | |
| Relationship established |  | Was a relationship established prior to study commencement? | 8 |
| Participant knowledge of  the interviewer |  | What did the participants know about the researcher? e.g. personal  goals, reasons for doing the research | 8,9 |
| Interviewer characteristics |  | What characteristics were reported about the interviewer/facilitator?  e.g. Bias, assumptions, reasons and interests in the research topic | 8 |
| **Domain 2: Study design** | | | |
| *Theoretical framework* | | | |
| Methodological orientation  and Theory |  | What methodological orientation was stated to underpin the study? e.g.  grounded theory, discourse analysis, ethnography, phenomenology,  content analysis | 7-9 |
| *Participant selection* | | | |
| Sampling |  | How were participants selected? e.g. purposive, convenience,  consecutive, snowball | 7 |
| Method of approach |  | How were participants approached? e.g. face-to-face, telephone, mail,  email | 7 |
| Sample size |  | How many participants were in the study? | 10 |
| Non-participation |  | How many people refused to participate or dropped | 9 |
| *Setting* | | | |
| Setting of data collection |  | Where was the data collected? e.g. home, clinic, workplace | 7 |
| Presence of non-participants |  | Was anyone else present besides the participants and researchers? | nil |
| Description of sample |  | What are the important characteristics of the sample? e.g. demographic  data, date | 7 |
| *Data collection* | | | |
| Interview guide |  | Were questions, prompts, guides provided by the authors? Was it pilot  tested? | 8 |
| Repeat interviews |  | Were repeat inter views carried out? If yes, how many? | 22 |
| Audio/visual recording |  | Did the research use audio or visual recording to collect the data? | 9 |
| Field notes |  | Were field notes made during and/or after the inter view or focus group? | 9 |
| Duration |  | What was the duration of the inter views or focus group? | 8 |
| Data saturation |  | Was data saturation discussed? | 9 |
| Transcripts returned |  | Were transcripts returned to participants for comment and/or correction? | 9 |
| **Domain 3: analysis and findings** | | | |
| *Data analysis* | | | |
| Number of data coders |  | How many data coders coded the data? | 9 |
| Description of the coding  tree |  | Did authors provide a description of the coding tree? | 9 |
| Derivation of themes |  | Were themes identified in advance or derived from the data? | 9 |
| Software |  | What software, if applicable, was used to manage the data? | 9 |
| Participant checking |  | Did participants provide feedback on the findings? | 9 |
| *Reporting* | | | |
| Quotations presented |  | Were participant quotations presented to illustrate the themes/findings?  Was each quotation identified? e.g. participant number | 25 |
| Data and findings consistent |  | Was there consistency between the data presented and the findings? | 9 |
| Clarity of major themes |  | Were major themes clearly presented in the findings? | 10-16 |
| Clarity of minor themes |  | Is there a description of diverse cases or discussion of minor themes? | 10-16 |

# **Interview topic guides**

## Policy makers

**WARM UP**: Welcome, review of ethics and of the IDI’s purpose and process.

1. What kinds of attitudes do people within Federal entities (for example, XXX) have toward refugees and asylum seekers (abbreviated to RAS)?
2. How do these attitudes *encourage* or *discourage* Federal entities (for example, XXX) from supporting the implementation of low-intensity interventions like PM+? (for example, through Cantonal integration settings)

- What do you think is missing when it comes to psychological or mental health supports?
- Do influential people at the top see a need for non-specialized/non-medical (community-based) care for RAS? Why/why not?

1. What are the preferences of representative decision makers (at the Federal level) when it comes to RAS integration in Swiss society?

- Do key leaders see a need for promoting mental wellbeing as part of this agenda? Please explain why/why not

1. What are some barriers to RAS integrating in Swiss society? What needs to change to remove or reduce these barriers?
2. What level of support (if any) is need from the Federal entities (for example, XX) to implement and deliver low-intensity interventions like PM+ across Cantonal settings?

- What are the political and economic conditions at the Federal level?
- How do these conditions enable or hinder the Federal entities (for example, SEM) from supporting PM+ implementation at a Cantonal level?

1. Are there screening practices in place to detect mental health issues at the Federal asylum centers? Do you see a need for this?

- How would such screening for mental health affect asylum and integration goals?

1. Is there a need for a centralized strategy (i.e., federally supported) to screen of for mental health problems in RAS?

- When mental health problems are detected within the Federal reception centers, what do you think needs to happen next support RAS?
- How would such an initiative operate? What level of coordination is needed (e.g., between Federal and Cantonal entities) to make such a strategy work?
- What are the barriers to implementing such a centralized strategy?

1. To what extent does XXX have established connections to other Federal entities?

Please describe these connections

1. To what extent do you network or exchange information with colleagues outside of the Federal system? (e.g., collaborations with Cantonal entities?) Please describe these connections

- How can these partnerships be leveraged for the purpose of supporting mental health initiatives for vulnerable groups (such as RAS) in Switzerland?

1. What kinds of Federal legislation, regulations, professional group guidelines/recommendations, or accreditation standards might help or hinder implementation of low-intensity interventions like PM+ in Switzerland?
2. Is there scope to integrate the delivery of low-intensity interventions such as PM+ as part of current integration policies for RAS?
3. What types of funding are needed from outside the Cantonal system to implement low intensity interventions like PM+?

- To what extent is this funding available?

1. What barriers do you see to adding low intensity interventions such as PM+ as part of mandatory health insurance?

- Do you a possibility for government-mandated health insurance to cover implementation of low-intensity intervention like PM+ for vulnerable groups like RAS? Why/why not?

1. What kinds of pressures outside of the Cantons may influence implementation of low-intensity interventions like PM+ within the Cantons?
2. To what extent is there pressure felt on a national level to improve RAS integration via target of mental health initiatives? Where do these pressures come from? [e.g., within Federal level, from Canton level/municipalities/communities?]
3. How can we overcome the Federal vs Cantonal divide when it comes to coordinating health promotion +prevention activities?

- For example, is there scope for Federal agencies to play a motivating role to cantons when it comes to promoting mental health initiatives within the integration agenda?

**WRAP UP:**

1. Is there anything else you want to share with me today?

## Specialists and SPIRIT organisational management

**WARM UP**: Welcome, review of ethics and of the IDI’s purpose and process.

1. To what extent are XXX providers aware of the mental health needs and preferences of the RAS residing in the country?

PROMPT: How "in touch" are staff and leadership with the needs of this population?

1. How well do you think a peer-based model of care like PM+ will meet the needs of RAS?
   - - In what ways will the intervention meet their needs?

What barriers will RAS face in participating in an intervention like this?

1. What kind of information or evidence are you aware of that shows whether or not peer-based models of mental heath care will work in the Swiss health system?

PROBE: Helper feedback, psychotherapist feedback, or scientific evidence

1. How do professional institutions perceive the implementation of peer-based psychological models of care?
2. What do influential stakeholders think of the intervention?
3. What are the barriers to implementation from the perspective of professional associations? How do you think we can overcome these barriers?
4. What kind of supporting evidence or proof is needed about the effectiveness of PM+ to get professionals (e.g., administration, other types of professionals – social workers, psychologists, nurses) on board?
5. What kinds of alterations do you think PM+ will need so it will provide the most benefit in Switzerland?
6. Who will decide (or what is the process for deciding) whether changes are needed to PM+ so that it works well in Switzerland?
   1. How will you know if it is appropriate to make any changes?
7. How would PM+ interface with other programs offered through canton of XX psychiatry services?

PROBE (only if relevant)

- Triage to PM+ prior specialist service or psychotherapy course
- Triage in the event of crises

1. Where do you see the provision of peer-based models of care like PM+ being integrated?
2. How does PM+ compare to other similar existing programs (i.*e., peer-based or low-level*) in the Swiss health system?
   1. What advantages does the intervention have compared to existing programs?
   2. What disadvantages does the intervention have compared to existing programs?
3. How does PM+ compare to other alternatives (i.e., *psychotherapist or psychiatrist led*) that may have been considered or that you know about?
   1. What advantages does the intervention have compared to these other programs?
   2. What disadvantages does the intervention have compared to these other programs?
4. Is there another type of intervention that people would rather implement for RAS?
   1. Can you describe that intervention?
   2. Why would people prefer the alternative?
5. What sort of advocacy do peer-based models of care need in order to receive the attention of policy makers? To get official support?
   1. From whom and why?
6. Are there people in your setting (e.g., may be other types of professionals you work with) who are likely to advocate (go above and beyond professional responsibilities) for the implementation of peer-based models of care like PM+?
   - - How do you think they can help with widespread implementation of PM+?

PROBE: What kinds of incentives and/or disincentives may influence implementation and/or delivery of PM+ within the canton of XX?

1. What level of endorsement or support have you seen or heard from leaders (i.e., influential clinicians, leaders etc) for peer-based models of mental health care?
   - - Who are these leaders and how has this affected things so far? Going forward?
2. What kind of support or actions can you expect from professional organizations to help make implementation of peer-based models of care successful?
   - - Who are these leaders? How do attitudes of different leaders vary?
     - Do they know about the intention to implement PM+?
     - What kind of support can you expect going forward? Can you provide specific examples?
     - What types of barriers might they create?
3. What are the different financing strategies available to support routine delivery of peer-based interventions such as PM+?

PROBE:

- Where do you see PM+ (exemplar low-intensity psychological model of care) fitting better, with regards to financing (in terms of coordination of care)?
  - Health, social or integration sector (if relevant)
- Would this be a federal or cantonal mandate to put this coordination of care into place?

1. What are the barriers to financing low intensity interventions such as PM+ within the canton?

- PROMPT: could PM+ be added?

1. What kind of quality assurance do low-intensity interventions (delivered by peers) need to meet to be funded?
2. Are there current peer-based treatment approaches that are funded by the health system/canton of XX? What criteria did they need to meet to get funded?

**WRAP UP:**

1. Is there anything else you want to share with me today?

## Non-specialist providers

**WARM UP**: Welcome, review of ethics and of the IDI’s purpose and process.

1. To what extent are local providers for RAS [i.e., social services, center for migrants, NGOs) aware of the mental health needs and preferences of the RAS residing in the country?

*(PROMPT: How "in touch" are staff and leadership with the needs for peer-based interventions like PM+?*

1. How well do you think the peer-based programs like PM+ will meet mental health needs of RAS?

*(Tell me more – in what ways?)*

1. What barriers will (or do) RAS face when participating in an intervention like PM+?
2. What have you head/learned about RAS experiences with PM+?
3. What do you think low-level interventions are is trying to do for refugees achieve in your community?  (i.e., what is the purpose)
4. What kind of evidence are you aware of that tells you whether or not PM+ will work for refugees in your community?  e.g., peer feedback, your own reading, your own experience

- Follow up: How does this influence your role as a helper?

1. How does your community perceive the PM+ intervention?
2. What do influential people in your community think of the interventions?
3. What are barriers from the perspectives of influential people in your community about widely implementing PM+ in the community?
4. How could PM+ be compatible with other programs offered for refugees in your community?

1. What are your suggestions when it comes to ***where*** PM+ delivered for refugees in the community?

- What sort of setting do you think is appropriate? Can you give examples

1. What kinds of alterations do you think leaders will need to make to ***whom and how*** PM+ is delivered so it will provide the most benefit in your setting?
2. Who should be able to decide how these changes are made to PM+ so that it works well in your setting? [e.g., non-specialists, specialists?]
3. In your opinion, what does a peer-based intervention like PM+ do for RAS, that a specialist driven intervention cannot (i.e., by a psychiatrist, psychologist)?
4. Are you aware of other peer-based programs been delivered to support the mental health of refugees?

- If yes, how do these compare to PM+?
- What are the advantages/disadvantages of PM+ relative to other alternatives?

1. Why do you think the SPIRIT project has chosen peers to deliver the intervention?

- What are the advantages?
- What are the disadvantages?

1. Are there any disadvantages to someone engaging in PM+ as a helper? *PROBE: examples*
2. How could helpers such as yourself advocate for peer-based interventions like PM+ to be routinely delivered across the country?

- What sort of community members or professionals or policy makers could you engage with to motivate them to consider putting in resources to routinely delivering PM+?

1. What challenges do you see when it comes to implementing a peer-based program (like PM+) in the long-term (beyond SPRIRT)?

- Follow up:
  How optimistic (or not) are you about the future of this program in Switzerland?

1. What level of endorsement or support have you seen or heard from leaders (i.e., influential clinicians, SPIRIT coordinators etc) for peer-based models of mental health care?
   - - Who are these leaders and how has this affected things so far? Going forward?
2. What kind of support or actions can you expect from professional, communal, social services organizations to help make implementation of peer-based models of care successful in the future?
   - - Who are these leaders? How do attitudes of different leaders vary?
     - Do they know about the intention to implement PM+?
     - What types of barriers might they create?
3. What do you believe helpers need in order to sustain themselves in this role in the long-term? *(Can you elaborate? provide examples)*

*What other forms of recognition would help you do you job as a helper better?*

Follow-up as relevant

- Do health professionals recognise these needs?
- Do social service providers recognise these needs?

1. What do you suggest can be done to better meet these needs you have described?

*(Tell me more – in what ways?)*

Can you describe any issues around being a helper that you would like more advice on or are unclear about?

**WRAP UP:**

Is there anything else you want to share with me today?

# **Coding: CFIR constructs and definitions**

| CFIR Domain | Implementation research construct from CFIR | Definition of construct |
| --- | --- | --- |
| Intervention Characteristics | Relative Advantage | Belief in the advantage of implementing the intervention versus another option. |
|  | Evidence strength and quality | Belief in the strength of evidence for the intervention’s effectiveness. |
|  | Complexity | Extent to which PM+ is complicated, which may be reflected by its scope |
|  | Adaptability | Belief that the intervention can be adapted, modified, or refined to better fit the organization, treatment providers, or patient needs. |
|  | Design quality and Packaging | Belief that quality assurance standards are known and can be met for programs in order to be funded |
| Inner Setting | General structural characteristics |  |
|  | Relational connections | The degree to which there are high quality formal and informal relationships, networks, and teams within and across Inner Setting boundaries (e.g., structural, professional). |
|  | Implementation climate | The health/integration systems’ capacity to make changes, including a shared vision of the need for improvement and commitment to the program’s growth. |
|  | Culture | Beliefs, values, ethics and basic assumptions within the system that welcome the intervention. |
|  | Implementation/delivery related constructs |  |
|  | Tension for Change | Belief that the current situation is intolerable and needs to change. |
|  | Compatibility | Belief in the relative compatibility of implementing the invention within broader services available to participants |
|  | Relative Priority | The belief that the intervention is a high priority for the program to implement |
|  | Incentive Systems | The degree to which tangible and/or intangible incentives and rewards and/or disincentives and punishments exist to support implementation and delivery of the intervention |
|  | Mission Alignment | The degree to which implementing and delivering the innovation is in line with the overarching commitment, purpose, or goals in the Inner Setting. |
|  | Readiness for implementation / Leadership support | The system is at the ‘action’ stage of implementing this intervention. Practices that have already implemented the intervention may be at the ‘sustainment’ stage – continuation of ‘action.’ |
|  | Available resources | The degree to which resources are available to implement and deliver the innovation. |
| Outer Setting | Patient needs and resources | Belief that the intervention fits with patient needs, preferences, and resources. |
|  | Local attitudes | The degree to which sociocultural values (e.g., shared responsibility in helping recipients) and beliefs (e.g., convictions about the worthiness of recipients) encourage the Outer Setting to support implementation and/or delivery of the innovation. |
|  | Local Conditions | The degree to which economic, environmental, political, and/or technological conditions enable the Outer Setting to support implementation and/or delivery of the innovation. |
|  | Partnerships and Connections | The degree to which the Inner Setting is networked with external entities including federal agencies, divisions, academic affiliations, and professional organization networks. |
|  | Policies and Laws | The degree to which legislation, regulations, professional group guidelines and recommendations, or accreditation standards support implementation and/or delivery of the innovation. |
|  | Financing | The degree to which funding from external entities (e.g., grants, reimbursement) is available to implement and/or deliver the innovation. |

# **Study groups overview**

The following study groups were interviewed in both individual interview or focus group discussion formats. Following an ecological approach, individuals were selected based on their involvement across multiple levels of Federal and/or Cantonal sectors as it related to refugee care and/or integration.

| Type of stakeholder | Participant Groups/Entities |
| --- | --- |
| *Policy makers and leaders* | Federal Office of Public Health  State Secretariat for Migration  Federal Quality Commission  Health Promotion Switzerland  Cantonal Psychiatry leaders  Cantonal Integration leaders |
| *Specialists* | Chief Doctors  Psychologists  Psychiatrists |
| *Organisational management and Implementers* | Swiss Red Cross  SPIRIT team managers |
| *Non-specialists* | Refugee peer providers of PM+ |

| Descriptions of select entities | |
| --- | --- |
| *Federal Office of Public Health* | Federal government body responsible national health care policy |
| *State Secretariat for Migration* | Federal government body regulating the conditions under which persons enter Switzerland |
| *Federal Quality Commission* | An independent non-parliamentary commission. It supports the Federal Council in developing the quality of medical services within the framework of the Federal Health Insurance Act |
| *Swiss Red Cross* | An independent humanitarian Organisation providing a broad range of services for socially disadvantaged and vulnerable communities in Switzerland. |
| *Health Promotion Switzerland* | Health Promotion Switzerland is a foundation established by the Federal Health Insurance Act of 1994. It is mandated through this legislation to initiate, coordinate, and evaluate policies to promote health and prevent disease. |

# **Coding tree**

| Domain | CFIR Construct | Sub-code |
| --- | --- | --- |
| Intervention Constructs | Relative Advantage | Facilitator |
|  | Evidence strength and quality | Barrier |
|  | Complexity | Barrier |
|  | Adaptability | Facilitator |
|  | Design quality and Packaging | Barrier/Facilitator |
| Outer Setting | Local attitudes | Barrier/Facilitator |
|  | Local Conditions | Barrier/Facilitator |
|  | Partnerships and Connections | Barrier/Facilitator |
|  | Policies and Laws | Barrier/Facilitator |
|  | Financing | Barrier |
| Inner Setting | Relational connections | Barrier/Facilitator |
|  | Implementation climate | Barrier/Facilitator |
|  | Tension for Change | Barrier/Facilitator |
|  | Compatibility | Facilitator |
|  | Incentive Systems | Barrier/Facilitator |
|  | Available resources | Barrier |
|  | Mission Alignment | Barrier/Facilitator |
|  | Readiness for implementation/implementation climate | Barrier/Facilitator |
| **Characteristics of Individuals** (inductive) | Individual stage of change (Professional Identity; scepticism) | Barrier |
| **Process** (inductive) | Planning | Facilitator |
|  | Engaging | Facilitator |

# **Deductive disclosure statement**

Given the small and identifiable nature of policy stakeholder groups within Switzerland's Federal and Cantonal health and integration sectors, measures were taken to minimise the risk of deductive disclosure. Accordingly, no identifying information (i.e., including age, gender, or specific occupational title) has been reported for individual participants. Stakeholder attributions are made at the group level only (e.g., Federal health leader, Cantonal psychiatrist) to preserve confidentiality whilst maintaining transparency about the source of reported perspectives.
